# Maxillary Mucormycosis Osteomyelitis in Post COVID-19 Patients: A Series of Fourteen Cases

**DOI:** 10.3390/diagnostics11112050

**Published:** 2021-11-05

**Authors:** Wael M. Said Ahmed, Amira M. Elsherbini, Nehal M. Elsherbiny, Mohamed El-Sherbiny, Nevine I. Ramzy, Ahmed F. Arafa

**Affiliations:** 1 Oral and Maxillofacial Department, Faculty of Dentistry, Mansoura University, Mansoura 35516, Egypt; drwaelmohamed2020@gmail.com; 2 Oral Biology Department, Faculty of Dentistry, Mansoura University, Mansoura 35516, Egypt; 3 Department of Biochemistry, Faculty of Pharmacy, Mansoura University, Mansoura 35516, Egypt; drnehal@mans.edu.eg; 4 Department of Pharmaceutical Chemistry, Faculty of Pharmacy, University of Tabuk, Tabuk 47512, Saudi Arabia; 5 Department of Basic Medical Sciences, College of Medicine, AlMaarefa University, Riyadh 71666, Saudi Arabia; 6 Department of Anatomy, Mansoura Faculty of Medicine, Mansoura University, Mansoura 35516, Egypt; 7 Pathology Department, Faculty of Medicine, Cairo University, Giza 11562, Egypt; ramzywebas@gmail.com; 8 Oral and Maxillofacial Department, Zagazig University, Zagazig 44519, Egypt; arafabelal@gmail.com

**Keywords:** COVID-19, mucormycosis, maxilla, osteomyelitis

## Abstract

During the current pandemic of COVID-19, numerous manifestations and complications have developed. Patients with COVID-19 are at high risk of fungal infections, such as mucormycosis, that may result directly from COVID-19 infection and/or as a side effect of the drugs used in COVID-19 treatment protocol, such as dexamethasone, hydroxychloroquine, and antibiotics. In this report, we described a series of 14 cases with maxillary mucormycosis osteomyelitis in immediate post-COVID-19 patients.

## 1. Introduction

Coronavirus disease 2019 (COVID-19 & COVID) is a contagious pandemic disease caused by severe acute respiratory syndrome coronavirus2 (SARS-CoV-2). COVID-19 has variable signs and symptoms, but often include fever, cough, headache, hypoxia, thromboembolic disorders, fatigue, dyspnea, diarrhea, anosmia, and ageusia [1]. About 1/3 of infected people are asymptomatic. In symptomatic patients, 81% have mild to moderate symptoms, while 14% develop severe symptoms and 5% suffer critical symptoms [2]. Immunocompromised and old people are at a greater risk of developing severe symptoms. Symptoms may occur 1–14 days’ post COVID-19 exposure and some patients continue to experience a variety of effects for months after recovery (long COVID) [3].

Osteomyelitis is an inflammatory condition affecting the bone that usually begins from the medullary spaces, rapidly involving the Haversian systems, and extending to periosteum of the involved area. Inflammation and edema that result from the infection compromise the blood supply leading to bone ischemia, which in sequence becomes necrotic [4].

Mucormycosis is an angio-invasive fungal infection caused by fungi in the order Mucorales and usually begins in the nose and para-nasal sinuses. Infection may result from inhalation, ingestion, or contamination of ulcerated mucosa or skin by fungal spores. It is rapidly progressing and potentially destructive and life-threatening in immunocompromised, debilitating, or diabetic individuals [5,6].

## 2. Case Series

**Figure 1:**
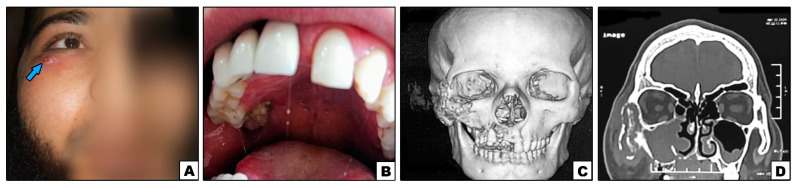
Case#2: (**A**) Extraoral swelling of the right side of the face (note: sinus tract inferolaterally to the eye {arrow}). (**B**) Exposed necrotic bone in the right posterior palate. (**C**,**D**) 3D and cronal CT showing bone destruction of right maxilla, inferior orbital rim, and lower part of zygoma.

**Figure 2:**
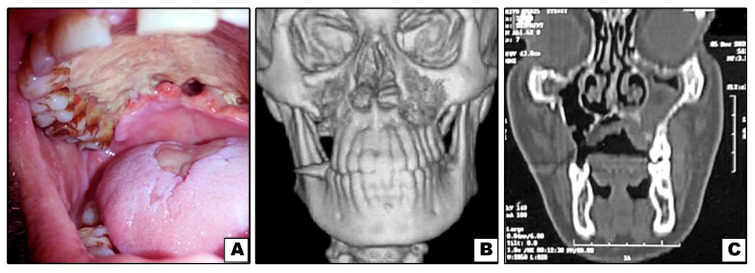
Case#3: (**A**) Exposed necrotic palatal bone with palatal perforations. (**B**,**C**) 3D and coronal CT showing bone destruction of right and left maxillae (not involving inferior orbital rims).

**Figure 3:**
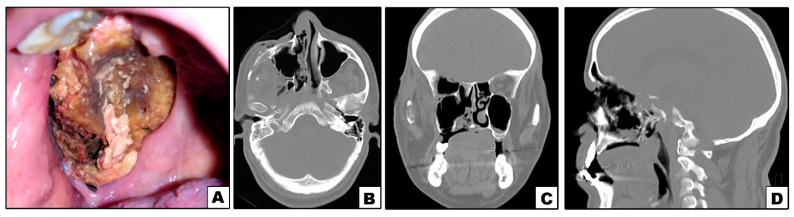
Case#5: (**A**) Exposed necrotic palatal and alveolar bone on right side. (**B**–**D**) Axial, coronal and sagittal CT showing bone destruction involving right maxilla and orbit.

**Figure 4:**
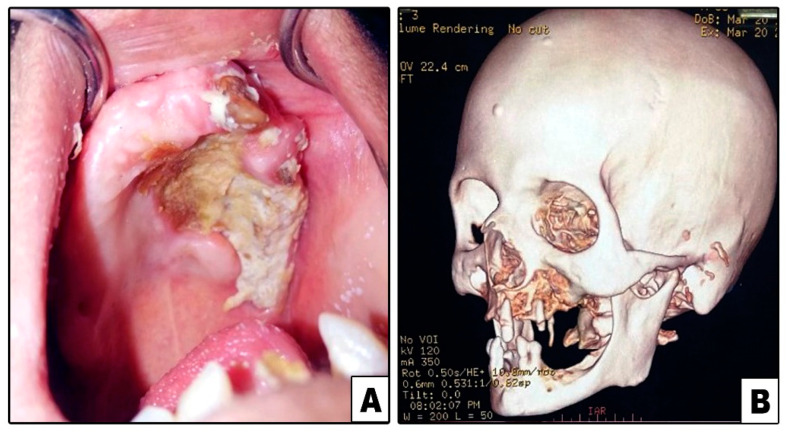
Case#7: (**A**) Exposed necrotic palatal and alveolar bone on left side. (**B**) 3D CT showing bone destruction involving left maxilla and lower part of zygoma.

**Figure 5:**
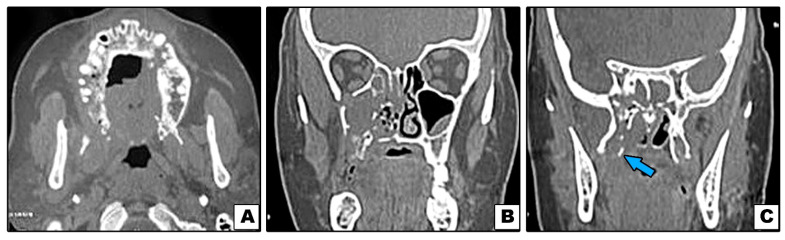
Case#13: (**A**–**C**) Axial and coronal CT showing bone destruction involving premaxilla, right maxilla with pterygoid plates (arrow), and not involving inferior orbital rim.

This case series included 14 patients and followed the Declaration of Helsinki guidelines, also it was approved by Ethical Committee of Faculty of Dentistry, Mansoura University. An informed consent was signed from each patient. All the patients had recent COVID-19 infection and were quarantined for 20–30 days; four patients in home, seven patients in hospital ward, and three patients in ICU. The patients attended Oral and Maxillofacial Surgery Departments in Zagazig University and Faculty of Dentistry of Mansoura University, Egypt, 14–30 days’ post COVID-19 recovery, complaining from pain, loss of one or more maxillary teeth, exposed bone, pus discharge, and bad odor. Clinical and radiographic examination were shown in Table 1 and Figure 1, Figure 2, Figure 3, Figure 4 and Figure 5. Swab for culture and incisional biopsy were taken from each patient.

**Figure 6:**
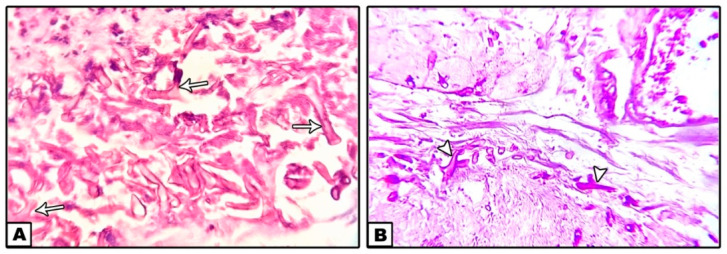
Nonseptate hyphae with right angle branching (arrows) and spores of mucormycosis surrounded by necrotic tissues and a dense inflammatory infiltration. ((**A**): H&E ×40, (**B**): PAS ×40).

Histopathological picture (Figure 6) revealed an invasive mucormycosis showing collections of nonseptate hyphae with right angle branching and spores of mucormycosis surrounded by necrotic tissues and a dense inflammatory infiltration.

All the patients received 150 mg/3 days of Fluconazole antifungal drug (Diflucan, Pfizer, Brooklyn, NY, USA) for 1 month, and 30 dives of hyperbaric oxygen (0.5 h/day). After that, the patients had surgical treatment varying from debridement and curettage to extensive bone removal according to the degree of bone involvement in each case. (Table 1) After surgery, all patients received 20 dives of hyperbaric oxygen (0.5 h/day), and 150 mg/3 days of Fluconazole was continued for 2 weeks.

## 3. Discussion

During the current pandemic of COVID, numerous manifestations and complications have occurred, including increased risk of fungal infections [8].

Mucormycosis is a fungal infection caused by fungi in the order Mucorales. It has a remarkable high morbidity and mortality, and its incidence is in the ascendant. In healthy individuals, growth of mucor spores is usually resisted by phagocytes, however, in immunocompromised individuals where the host response is compromised, infection exacerbates. The mucor hyphae have an affinity to blood vessels, and invade them, proliferate, and spread within the vessel walls, causing a series of events such as thrombosis, ischemia, necrosis, and finally sequestration of the involved tissue [9].

Mucormycosis occurs after the inhalation of the fungal spores and invasion of the paranasal sinuses, causing necrosis of the nasal mucosa, turbinates, and palate. If untreated or unnoticed, the disease has the ability to spread through the whole face, resulting in facial bone necrosis and penetration of orbits and cranium causing mortality. Osteomyelitis of the maxilla is usually rare due to its rich blood supply and presence of thin cortical plates, however, the high angio-invasiveness potential of mucor fungi affects the endothelial lining of blood vessels, causing vascular insufficiency and bone necrosis resulting in mucormycosis osteomyelitis [10].

Mucormycosis risk factors include immunosuppressive conditions, diabetes mellitus, depilated patients, and leukemia. In our cases, nine patients were diabetics before quarantine, and five patients were not diabetics but showed temporary hyperglycemia after COVID-19 quarantine (cases#1, 2, 8, 11 and 13). Acidosis and hyperglycemia in diabetic patients lead to suppressed phagocytic capacity of granulocytes, deteriorated antioxidant system, and increased serum free iron, favoring fungal growth and proliferation [11].

Patients with COVID-19 infection may suffer from hypoxia, and during quarantine they can become malnourished and debilitated, resulting in disruption of their immunity [12]. Also, COVID-19 is known to have the ability to cause thromboembolism, which may result in closure of blood vessels, ischemia, and subsequently tissue necrosis [13]. Furthermore, it has been reported that some individuals with COVID-19 develop a diabetes-like syndrome [14].

In Egypt, treatment protocol of COVID-19 contains some drugs that may enhance fungal infection (mucormycosis), such as dexamethasone 1.5 mL/24 h, hydroxychloroquine 400 mg/12 h, and broad spectrum antibiotic (cefotax) of 1 gm/12 h during quarantine period.

In addition to the main effects of corticosteroids as anti-inflammatory and immunosuppressive, steroids can cause drug-induced hyperglycemia. They not only exacerbate hyperglycemia in patients with known diabetes mellitus, but also induce diabetes mellitus in patients without documented hyperglycemia with an incidence that can reach up to 46% of patients (that can explain post-COVID-19 hyperglycemia in patients with no history of diabetes before quarantine), and increases in glucose levels up to 68% compared to baseline as they suppress the effectiveness of insulin (insulin resistance) and induce the liver to release stored glucose into the blood stream [15].

Hydroxychloroquine is mainly an anti-inflammatory and immunosuppressive drug used for treatment of malaria, rheumatoid arthritis, and lupus. Therefore, it can decrease patients’ immunity and consequently enhance fungal growth [16]. Furthermore, prolonged use of broad-spectrum antibiotics may cause elimination of the competitive influence of the normal bacterial flora resulting in fungal overgrowth [17].

## 4. Conclusions

From this case report we concluded that mucormycosis is a serious condition that can result in high morbidity and mortality, and its incidence is on the rise, particularly in diabetic patients. COVID-19 infection plays a significant role in maxillary mucormycosis osteomyelitis either directly or as a side effect of COVID-19 treatment regimen.

## 5. Recommendations

We recommend careful monitoring of blood glucose level in COVID-19 patients during quarantine. As early diagnosis of these serious conditions is the key to allow for optimum treatment and better outcomes, careful and thorough examination of the oral and maxillofacial area should be done periodically in quarantined patients. Finally, systemic and local antifungal drugs should be prescribed to quarantined COVID-19 patients, particularly in diabetic and immunocompromised patients.

## Figures and Tables

**Table 1:** Personal data, clinical presentation, radiological features, and treatment for cases series.

Case No.	Age/ Gender	Medical Status	Site	Clinical Presentation	Radiographic Features(CT)	Treatment *
1	45/ Male	Post-COVID-diabetes	Alveolar bone + palatal bone anterior region	palatal bone exposure, teeth loosening	Palatal and alveolar bone rarefaction	Debridement and curettage
2	35/ Male	Post-COVID-diabetes	Right posterior palatal bone	ENB, extraoral pus discharge	BD involving right maxilla, inferior orbital rim, lower part of zygoma	Right total maxillectomy without orbital excenteration + resection of involved part of zygoma
3	65/ Male	Diabetic	Bilateral palatal + alveolar bone	ENB, PP	BD involving right and left maxillae (not involve inferior orbital rims) + left pterygoid plates	Bilateral subtotal maxillectomy + removal of left pterygoid plates
4	48/ Male	Diabetic	Bilateral palatal + alveolar bone	ENB, PP	BD involving right and left maxillae (not involve inferior orbital rims)	Bilateral subtotal maxillectomy
5	76/ Male	Diabetic	Right side of the palate + alveolar bone	ENB, PP, loss of vision	BD involving right maxilla and orbit	Right total maxillectomy with orbital excenteration
6	55/ Female	Diabetic	left side of the palate	ENB, PP	BD involving left maxilla	left subtotal maxillectomy
7	61/ Female	Diabetic	Left side of the Palate + Alveolar bone	ENB, loss of vision	BD involving left maxilla and lower part of zygoma	Left subtotal maxillectomy + resection of involved part of zygoma
8	45/ Male	Post-COVID-diabetes	Alveolar bone Anteriorly + palatal bone anterior region	palatal bone exposure, teeth loosening	Palatal and alveolar Bone Rarefaction	Debridement and curettage
9	52/ Male	Diabetic	Anterior third Of the palate	palatal bone exposure + PP	Palatal and alveolar Bone rarefaction+ ONC	Partial maxillectomy
10	53/ Female	Diabetic	Anterior third of the palate + alveolar process of anterior teeth	Necrosis of of premaxilla + PP	BD of premaxilla + ONC	Partial maxillectomy
11	29/M	Post-COVID-19 diabetes	Premaxilla + left palate	ENB of premaxilla and Left palate with alveolar processes	BD involving Premailla+ left maxilla (not involve inferior orbital rims)	Left subtotal maxillectomy
12	77/M	Diabetic	Bilateral palatal + alveolar bone	ENB and PP	BD involving right and left maxillae (not involve inferior orbital rims)	Bilateral subtotal maxillectomy
13	49/F	Post-COVID-19 diabetes	Anterior and right part of the palate + alveolar bone	ENB and PP	BD involving Premaxilla + ONC+ right maxilla with pterygoid plates (not involve inferior Orbital rim)	Right subtotal maxillectomy+ removal of right pterygoid plates
14	69/M	Diabetic	Right side of the Palate	ENB and PP	BD involving right maxilla (not involve inferior Orbital rims)	Right subtotal maxillectomy

Post-COVID-19-diabetes (patients were nondiabetic then became diabetic Post-COVID), Exposed necrotic bone (ENB), Bone destruction (BD), Palatal perforation (PP), oronasal communication (ONC). * According to Cordeiro’s [7] maxillectomy classification: Type I, Partial 1–2 maxillary walls; Type II, Subtotal of three to five maxillary walls including the palate; Type III, Total Involves all six maxillary walls: (a) Lacking orbital exenteration, (b) With orbital exenteration; Type IV, Radical includes five maxillary walls, palate and orbit excepted.

## Data Availability

Data are available upon request from the author.
